# Some Points of Interest in the Surgical Department of the Municipal Hospital at Frankfort

**Published:** 1903-05-02

**Authors:** 


### HOSPITAL ADMINISTRATION.

### CONSTRUCTION AND ECONOMICS.

### SOME POINTS OF INTEREST IN THE SURGICAL DEPARTMENT OF THE MUNICIPAL HOSPITAL AT FRANKFORT.

BY A CORRESPONDENT.

It is not my intention to describe fully the structure, working, etc., of this admirable modern hospital, but only several details which struck me as specially excellent during my recent visit. The surgical division is one of the three main blocks of the municipal hospital, which occupies a vast area on the outskirts of the town, and adjoins the beautiful woods which extend pretty well without interruption from the south bank of the river Main to Switzerland. In Germany, all new hospitals are being erected away from the centre of the towns, and in Frankfort a very extensive and well-organised ambulance service with numerous stations in the populated districts, establishes the intercourse between centre and periphery. The stations are in charge of resident doctors who are seconded by a well-trained ambulance staff, and the four-wheeled horse ambulance carriages are all that the most exacting medical man's mind can desire. The smart, light vehicles with the sign of the red cross are known all over Frankfort, and everybody, cart-drivers and all, make room when the bell of the conductor sounds. The hospital contains 760 beds, and is divided into three main parts: surgical, medical and skin; in addition there are a number of small blocks for the various infectious diseases. The pavilion system has been adopted throughout—even administration, kitchen, laundry engines, occupy separate blocks each. The meals are conveyed to the different stations by means of heated waggons.

#### The Wards.

Each ward building is surrounded by a garden, which is divided into two sections, one for women and one for men. In addition all the wards have roomy balconies, large enough to allow the beds to be wheeled out. In the surgical block these balconies run the length of the whole ward on both sides. All dirty linen, soiled dressings, and other refuse are sent away from these balconies, nothing of this kind being allowed to be kept in the ward lavatories. The wards are very light and wide, with windows and balconies on two sides, facing each other. The white enamelled beds—this is a special point—are placed about three feet from the wall of the ward, in which everything is accessible, every corner rounded off, everything smooth and of a washable material. Lockers, of enamelled iron and glass, only contain one small drawer; no part of a patient's clothing is kept in the wards; towels and washing necessaries are relegated to the bath-rooms. The hospital supplies all wearing apparel, including clothes for the patients who are up and about. A special linen room for each ward contains all these items. The floors are of terrazzo, and warmed by hot pipes which run underneath; an additional overground steam-heating system supplies more heat. The walls are covered with a hard, highly-varnished paint. The usual ward appliances are stored in a case in the centre of the ward. This is entirely of enamelled iron and glass, and has a large partition, about 5 feet long, 3 feet wide, and 2 feet high below, and one about half the size at the top, and this part contains the medicines, thermometers, and a few instruments. The poisons are locked up in a special case under the head sister's charge in the day-room. The doctors' washstands are fitted with porcelain basins and treadle taps, all pipes being well away from the wall, hence freely accessible for cleaning purposes. Every surgical ward also possesses a fixed instrument steriliser, which is worked by steam; this steriliser, however, is scarcely ever used, as dressings are not done in the ward, but in the special dressing-rooms belonging to each ward. These will be spoken of directly. Small portable desks which can be fitted to the foot of the beds for note taking, and a few enamelled chairs complete the bulk of the ward furniture. English nurses will miss flowers and plants in my description, but these are not in favour with German doctors for decorating the wards themselves, yet a goodly show of palms and flowers, however, is found in the adjoining day-room, a lofty, light apartment, with a lovely view on the grounds. Here convalescent patients sojourn, take their meals, play games, and look very happy and cheerful, which acts as a relieving contrast to the oppressiveness which one cannot help feeling in walking through the somewhat austere wards. Patients who are allowed to wash themselves, use the large bath-rooms, which are provided with rows of porcelain basins with hot and cold water. The use of the tooth-brush is obligatory. There is nothing worth special notice in the line of baths, lavatories, ward kitchens; everything is good, practical, and clean.

#### Surgical Dressing-Rooms.

The one thing which struck me as excellent and worthy of imitation is the special room for doing dressings. It owes its existence, of course, to the idea that in a ward dressings cannot be carried out with the detailed cleanliness required by the laws of aseptic surgery. But, quite deducting that primary cause, the humane side of this arrangement must appeal to everybody. Even with all care and delicacy in a ward, with screens, etc., the dressing hour is always a time of extreme and painful tension to every poor patient, and the sight of wounds—soiled dressings—which occasionally cannot be avoided altogether, produces a sensation of horror in many of the fellow sufferers. In this hospital, patients are spared this additional agony, and the transit from the ward to the dressing-room is effected with ease, the beds being fastened to india-rubber tyred bed-carriages, and the floors of the wards, landings, and dressing-rooms all being on the same level, with no raised portions interposed at the thresholds. The dressing-rooms are fitted like small theatres, with sterilisers and all the rest of a modern aseptic equipment, and the same disinfecting process of hands and arms, as in the operating theatre, is gone through, overalls worn, etc. I saw several children on their way to be dressed, and they all seemed perfectly happy, in fact, enjoying their ride thoroughly. When I compared their condition to that of our poor little patients, who see dressing after dressing, and the dread apparatus moving nearer and nearer to their own beds, I could not help feeling that in this one respect the Frankfort children were considerably better off.

#### The Operating Theatre.

The most wonderful part of the hospital is, without doubt, the operating theatre. Professor Rehn has spent years of thought on the working out of every minute detail of this his pet place, and the energy with which he succeeded in getting his plans carried out, is only equalled by the unique munificence of the town fathers who supplied the necessary funds, and are still doing so at every new suggestion of their pet surgeon, so as to make and maintain the place perfect, and Rehn's results what everybody in Germany knows them to be. To many, perhaps, the amount of detail observed at this clinic may seem absurd, but Rehn justly maintains that aseptic surgery can only be undertaken with this amount of detail and with a hospital built according to aseptic ideas, and that without such an apparatus nobody can and should venture to leave the broad and easy paths of older methods. Everything in this theatre is smooth, round, clean and simple. The eight wash-basins are of porcelain with treadle taps, and supplied with a glass shelf, containing the sterilised nail-brushes, instruments for cleaning and trimming nails, liquid potash soap, and a sand clock, timed for five minutes. The water, which is discharged through a rose tap, is regulated at a continual temperature of 39° Centigrade. The floor and walls are of white tiles. The operating table stands in a bow or semi-circle, which is built out from the main body of the theatre, the walls of this recess are almost entirely of glass sheets, and a skylight adds brightness. The glass instrument stands and basins are of the usual type, also the sterilisers, only the great advantage is that all the heating is done by turning on steam into the apparatuses, no gas or matches being used in any part of the building, except in the special rooms, where the gas and mercury regulators are placed for maintaining temperatures of fluids. The operating table from Stille, in Stockholm, is nickel-plated, and stands on a solid middle basis, not on wheels, but can be raised to any height and turned to every angle possible by moving very smoothly-working handles. It can be tilted to the right and left, the foot and the head ends can be raised, and two shoulder clasps allow the patient to be almost stood on his head without slipping. Any of us who have seen a bad case of theatre collapse where this position is so useful and yet so difficult to maintain, while all hands are wanted for many things, will appreciate this detail which economises about eight strong arms.

(*To be continued.*)

